# Ethical Challenges and Opportunities at the Intersection of One Health and Open Science in India: A Scoping Review

**DOI:** 10.12688/openreseurope.21085.2

**Published:** 2026-01-27

**Authors:** Naheeda Hamza, Rosemarie De La Cruz Bernabe, Uma Kulkarni, Alina Coman

**Affiliations:** 1 PhD Fellow, Centre for Medical Ethics, University of Oslo, Oslo, Norway; 2 Tutor, Centre for Ethics, Yenepoya (Deemed to be University), Mangalore, India; 3 Professor, Centre for Medical Ethics, University of Oslo, Oslo, Norway; 4 Professor, Department of Ophthalmology; Faculty, Centre for Ethics, Yenepoya (Deemed to be University), Mangaluru, Karnataka, India; 5 Associate Professor, Department of Nursing and Health Promotion, Oslo Metropolitan University, Oslo, Norway

**Keywords:** One Health, Open Science, Citizen Science, Data Sharing, India

## Abstract

**Background:**

Open Science (OS) aims at accelerating responsible scientific research, promoting transparency in data sharing, and ensuring that data is accessible to all citizens. On the other hand, One Health (OH), an interdisciplinary field addressing outbreaks of zoonotic infectious diseases, relies heavily on data sharing. Considering the current emphasis on OS practices, it is crucial to identify the challenges associated with practicing OS within the multi-sectoral framework of OH. This scoping review explores how OS principles intersect with issues of data ownership, privacy, and participation within India’s multi-sectoral OH research ecosystem.

**Objective:**

This study aims to identify the potential challenges and opportunities at the interfaces of OH, OS, society, and policy.

**Methods:**

We conducted a scoping review, adhering to the Preferred Reporting Items for Systematic Reviews and Meta-analyses extension for Scoping Reviews (PRISMA-ScR) guidelines. We searched PubMed, Web of Science, Embase (OVID), MEDLINE (OVID), and Global Health to identify the articles. We included original articles and policy briefs on OS, Citizen Science, and OH published between 2013 and 2023. The protocol was preregistered in the Open Science Framework (OSF).

**Results:**

A total of 46 studies met the inclusion criteria. We reviewed 33 original articles, 4 perspectives, 2 commentaries, 2 case studies, 2 policy briefs, 2 protocols, and 1 case report. Key challenges include barriers to cross-sector collaboration, data sharing, weak inter-ministerial collaboration, and challenges in Citizen Science. The study also highlights significant opportunities for advancing OH through improved data sharing techniques, and enhanced collaborative efforts.

**Conclusion:**

Addressing these challenges and opportunities may foster effective collaboration, ethical data sharing in OH. These strategies are crucial for advancing OH framework and improving health at the human-animal-environment interface in India and other low-and middle-income countries. This review underscores the importance of integrating OS for the sustainable development of OH initiatives in these settings.

## Summary of Revisions

This manuscript has been revised in response to the constructive feedback provided by reviewers 2 and 3. A few minor corrections were made to improve methodological transparency and overall clarity. First, a justification has been added for limiting the literature search to 10 years. This explains that the restriction was applied to ensure the inclusion of current policies, infrastructure, and implementation practices in OH. Additionally, we have cited the Manhattan Principles for OH (2004) for more clarity on the inclusion criteria. We have also explained how the India-specific criterion for article inclusion was applied during the screening process.

**Figure 1.0.**
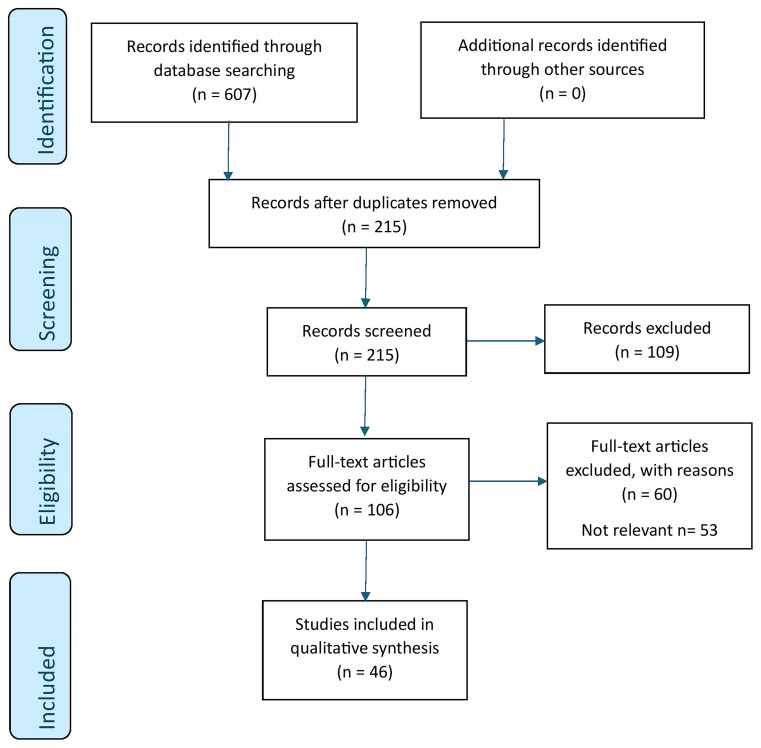
PRISMA schematic representation of the review process.

As suggested by reviewer 2, a sentence on page 10 has been corrected to improve clarity and and minor typographical errors have been addressed on pages 9,10, and 14. In response to reviewer 3, the word “authors” has been added on page 4 to improve grammatical correctness, and the reference to “ Fig 1.0” has been removed from the sentence on page 5. All revisions were made in accordance with the reviewers’ suggestions, and no major changes were made to the results or conclusions. Please note that these revisions do not alter the study’s findings but improve readability.

## Introduction

Open Science (OS) is defined as:

*``An inclusive construct that combines various movements and practices aiming to make multilingual scientific knowledge openly available, accessible and reusable for everyone, to increase scientific collaborations and sharing of information for the benefits of science and society, and to open the processes of scientific knowledge creation, evaluation and communication to societal actors beyond the traditional scientific community. It comprises all scientific disciplines and aspects of scholarly practices, including basic and applied sciences, natural and social sciences and the humanities, and it builds on the following key pillars: open scientific knowledge, open science infrastructures, science communication, open engagement of societal actors and open dialogue with other knowledge systems."* ^1^

OS focuses on making research processes and results accessible not only to professionals but also to citizens. As such, OS brings about a shift in traditional science, by providing a new framework on how to do science, and with it, new collaborative tools ^2^. By opening science, it also alleviates the barriers to working with transdisciplinary, collaborative, and multisectoral teams, such as in One Health (OH).

OH is “a multi-sectoral and integrated approach that aims to sustainably balance and optimize the health of people, animals, and ecosystems” ^3, 4^. Because OS is geared towards greater engagement and collaboration, increased and earlier sharing of scientific knowledge, and inclusion of other epistemological systems, there are very good reasons for OH researchers to practice OS. Practicing OH in an OS environment accelerates innovation, democratizes knowledge, and builds public trust. In the past decade, there has been a rise of infectious diseases globally. Approximately 75% of emerging and re-emerging diseases are of zoonotic origin. Due to this, almost all countries have adopted the OH approach by fostering collaboration and communication among the stakeholders working around the boundaries of the human-animal-environment interface ^3, 5^.

Providing early access to research results, fostering community collaboration and citizen science, promoting open tools and software, and preregistering studies are all vital for strengthening disease surveillance, informing policy decisions, and facilitating global cooperation. This approach makes India's health system more efficient, responsive, and globally integrated. Moreover, the use of FAIR (Findable, Accessible, Interoperable, and Reusable) data along with collaborations outside the academic community and other epistemic backgrounds may improve climate-health policies, and foster innovation.

However, due to the involvement of multiple stakeholders in OH such as epidemiologists, Veterinarians (Vets), ecologists, and policymakers, researchers in India face constant challenges in evaluating the impact of OH. Although the OS movement in research and innovations came into existence a decade ago, the openness of data sharing in OH research is not always executed ^3^. On account of the lack of aiding policies and low institutional capacities for OH in India ^5, 6^, we anticipate a wide range of challenges in incorporating OS principles into OH research. No scientific advancement is without challenge, and one such is the OH framework, which needs close attention to ensure ethics and research integrity in the era of OS. This article explores through a scoping review, the opportunities and challenges at the interfaces of OH, OS, society and policy.

## Methods

This Scoping review (ScR) was conducted as per the Joanna Briggs Institute (JBI) template for ScR ^7^ for a period of eight months (October 2023–May 2024). We also followed the JBI Manual for Evidence Synthesis for ScR. The screening of the articles was carried out through *Covidence* ^8^. The search results and the inclusion process is reported in the final scoping review and presented in a Preferred Reporting Items for Systematic Reviews and Meta-analyses extension for scoping review (PRISMA-ScR) ^9^. See Figure 1.0.

**Protocol and registration:** The proposal is registered in advance in the Open Science Framework- https://osf.io/zqw4c. The study was approved by the Yenepoya Ethics Committee-2 (YEC2/2023/044), India.

**Data sources and search strategy:** The search strategy aimed to locate published studies and grey literature. We searched PubMed, Web of Science, Embase (OVID), MEDLINE (OVID), and Global Health to identify the articles. NH conducted the search with the help of a Librarian at the University of Oslo. The keywords in the titles and abstracts of relevant articles were used to develop a full search strategy. The reference list of all included articles was screened for additional studies. Restricted-access articles were accessed through the University of Oslo online library. The search strategy is presented in ( Table 1.0). Since there were a limited number of articles on the Indian context, multiple keywords were applied, particularly in PubMed, to ensure a comprehensive search.

**Table 1.0.** Search strategy and articles retrieved.

Database	Keywords	Articles retrieved
**PubMed** **<2013 to April 30 2024>**	"One Health Challenges"[All Fields] AND India ("One Health" AND "Open Science") ("Public Health Research" AND "Open Data") ("One Health" AND "Policy Implications" AND India) ("One Health" AND "Data Sharing" AND India) ("One Health" AND Collaboration AND India) ("One Health" AND "Citizen Science" AND India) (("Community-Based Research Challenges" OR "Citizen Science Research") AND India)	182
**Web of Science** **<2013 to April 30 2024>**	("One Health Challenges") ("One Health" AND "Open Science") ("Public Health Research" AND "Open Data") ("One Health" AND "Policy Implications" AND India) ("One Health" AND "Data Sharing" AND India) ("One Health" AND Collaboration AND India) ("One Health" AND "Citizen Science" AND India) (("Community-Based Research Challenges" OR "Citizen Science Research")) AND India), ("Citizen Science" AND "Public Health Research" AND India)	125
**Embase** **Classic+Embase <2013 to November 07, 2023 >**	(Open science or Open access or Open data or Open publish* or open peer review or open education or open research or open tools or open methodology or Crowdsourc* or (data adj3 shar*)).tw,kf. ((Citizen or Volunteer or Community based or Consumer-Driven) adj2 (science or research*)).tw,kf. citizen science/ or exp open access/ use emczd	135
**Ovid Medline** **(R) ALL <2013 to November 08, 2024>**	(Open science or Open access or Open data or Open publish* or open peer review or open education or open research or open tools or open methodology or Crowdsourc* or (data adj3 shar*)).tw,kf. ((Citizen or Volunteer or Community based or Consumer-Driven) adj2 (science or research*)).tw,kf. citizen science/ or exp open access/ use emczd. Community-Based Participatory Research/ or Open Access Publishing/ use medall	115
**Global Health** **< 2013 to November 30, 2024>**	(Open science or Open access or Open data or Open publish* or open peer review or open education or open research or open tools or open methodology or Crowdsourc* or (data adj3 shar*)).tw. ((Citizen or Volunteer or Community based or Consumer-Driven) adj2 (science or research*)).tw. (one health* or onehealth*).tw.	50
**Summary**	Total number of articles	n = 607
	After deduplication	n = 215

### Eligibility criteria

**Inclusion of articles:** Original articles and policy briefs on OS, Citizen Science, and OH published in the last 10 years (2013–2023) in India are included. Commentaries, case studies, perspectives and policy briefs are also included for a comprehensive understanding of the topic. The search was limited to publications from the last 10 years. Although foundational concepts relevant to OH and OS predate this period, such as the Manhattan Principles for OH, 2004 ^10^, and early articulations of OS, this review aimed to capture articles reflecting on current policy, digital infrastructures, and implementation practices. 

Studies were included if they focused on India as the geographic or institutional context of the research, regardless of the country of journal publication. Articles authored by Indian researchers, or those examining Indian systems, policies, or population, were eligible even when published in international journals. During screening, eligibility was determined based on the study setting and content, such as references to India in the title, abstract, or full text.

**Exclusion of articles**: Duplicates, articles that did not mention OH, Citizen Science, OS and India, and articles whose full text could not be accessed are excluded.

### Title and abstract screening

The screening of abstracts on Covidence involved three steps. First, the title and abstract screening was carried out by the authors NH and AC. Second, the abstracts were promoted for full-text screening and were assessed in detail against the inclusion criteria. This was performed by NH, AC and UK. Finally, RB assessed all the included articles based on the inclusion and exclusion criteria and decided on unresolved conflicts.

### Data extraction and charting

Of the 607 studies identified through database searching, 392 articles were found to be duplicates. All the articles identified are uploaded into *EndNote Software*, and the references were uploaded to *Covidence* ^8^. Based on the inclusion criteria, 60 studies were excluded as 23 were found to be non-relevant to the objectives of the study, and 29 were not in the Indian context. Therefore, a total of 46 studies were included for the review. Reasons for the exclusion of articles that do not meet the inclusion criteria are reported in Figure 1.0. We reviewed 33 original articles, 2 policy briefs, 2 commentaries, 4 opinion pieces, 2 case studies, 2 protocols, and 1 case report. The data was extracted from articles by NH using a data extraction form (Extended data). AC assessed the accuracy of the data extracted. The extracted data included author, year, methods, key findings, and the implications for OH.

### Data synthesis

The data was then coded using NVivo version 14.23.2 ^11^. A thematic coding approach was employed to extract and analyze the data in NVivo, guided by Braun and Clarke’s methodology ^12^. This process involved familiarizing ourselves with the data, generating initial codes, identifying themes, reviewing, and naming themes. Extracts relevant to the research question were coded, starting with open coding. These open codes were then grouped into categories and sub-categories to identify broader patterns ^13^. NH conducted the initial coding of the articles, while AC face-validated the coding by cross-checking citations linked to themes and analyzing the articles to ensure consistency and identify any major differences in coding. Since ethical issues embedded in OH were not specifically mentioned in the articles, we chose to synthesize codes into two overarching themes: challenges and opportunities in the implementation of OH within the context of OS.

## Results

A total of forty-six articles were considered for coding and thematic analysis. The findings highlight the challenges and opportunities in the implementation of OH within the OS framework in India.

**Figure 2.0.**
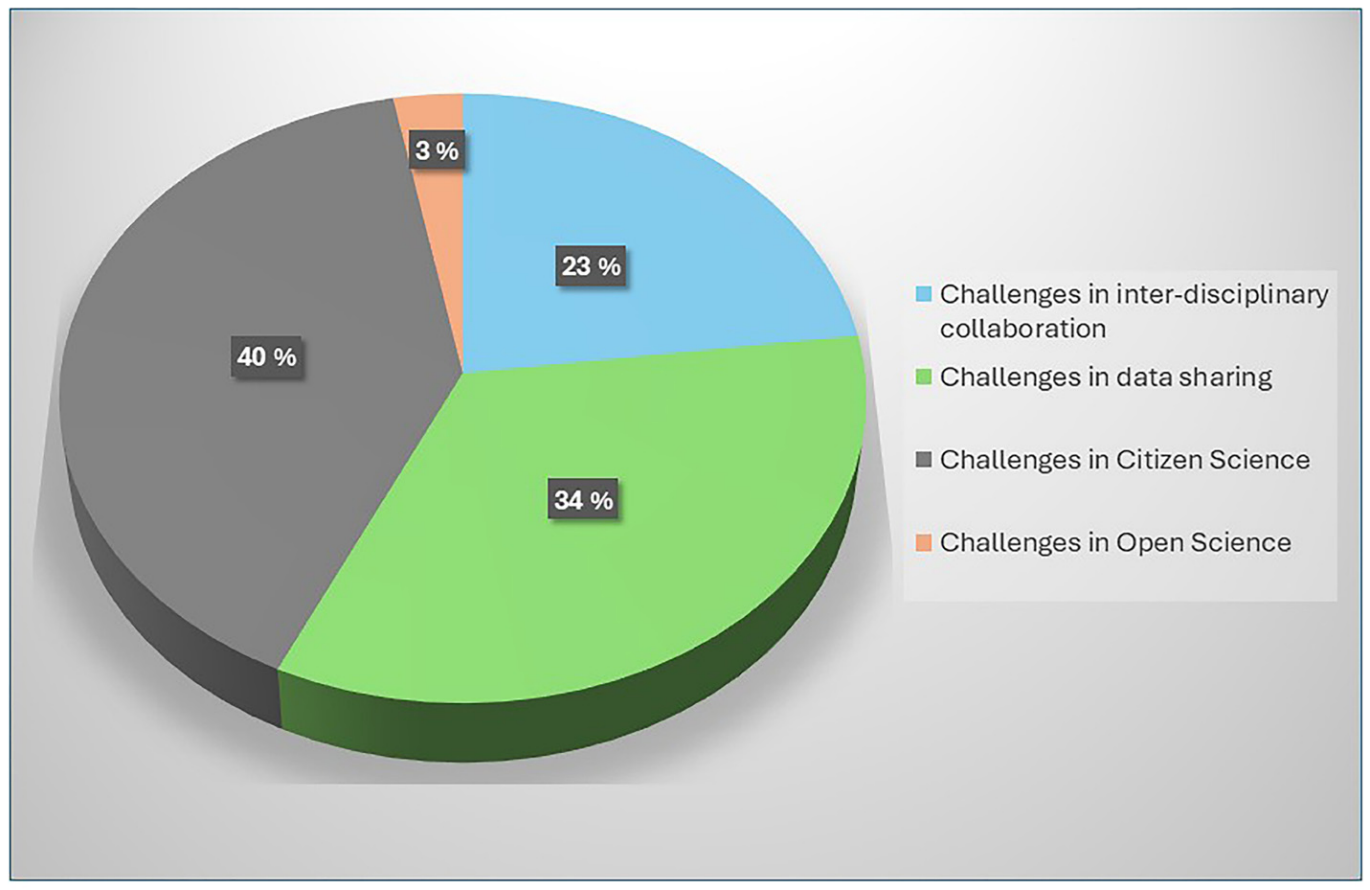
Distribution of challenges and the number of papers.

**Figure 2.1.**
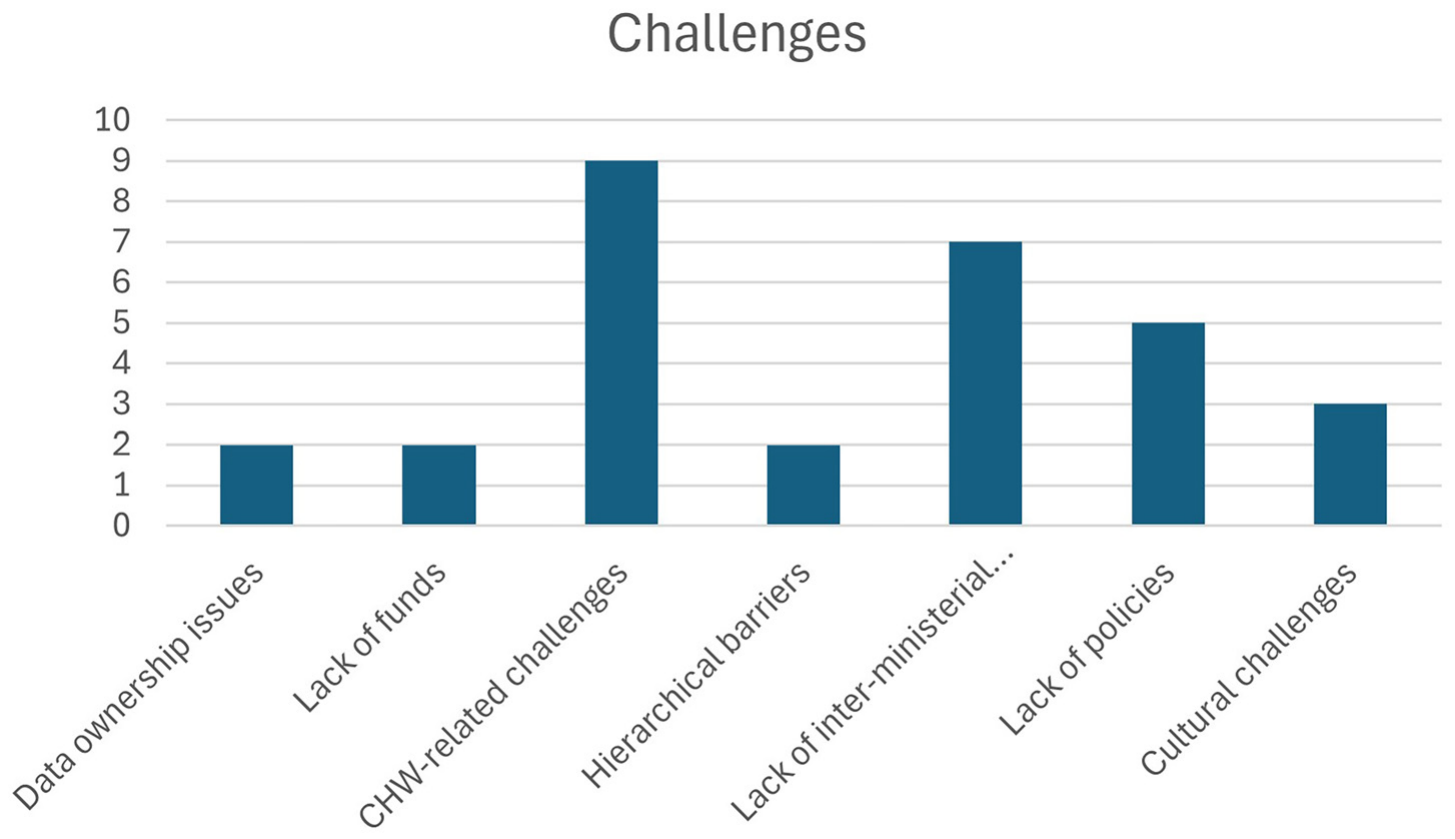
Distribution of categories and the number of papers.

**I. The challenges identified are categorized into two levels:**

a. ***Challenges in the implementation of OS in OH at Institutional level*** – While OH in India is still implemented at the national level, challenges at the institutional level are less prominent compared to national-level issues. Therefore, they are briefly described in this review.
b. ***Challenges in the implementation of OS in OH at National level-*** Given that OH initiatives in India operate at a national scale, greater emphasis is placed on the challenges encountered at this level. A total of eight categories of national-level challenges are identified, which are listed in Table 3.0. Also see Figure 2.0 and Figure 2.1.

**Table 3.0.** Challenges in incorporating Open Science at the Institutional and National level.

Categories	Sub-categories	Codes	Authors
** Institutional level**		*Limited infrastructure, limited funds, inadequate training, unclear reporting systems*	*Prejith *et al*., 2022, Taaffe J *et al*., 2023, Hoque M N *et al*., 2022, Yasobant S *et al*., 2019*
**National level**	Barriers to cross-sectoral collaboration	*Cultural challenges, different work culture, Community health worker-related challenges,*	*Asaaga *et al*. 2021, Bhat P *et al*. 2021, Yasobant S *et al*. 2020, Prejit *et al*. 2022, Wai-Looho C 2022*
	Challenges in data sharing	*No official strategies to facilitate data sharing, Confidentiality issues in data sharing*	*Prejit *et al*. 2022, Bhat P *et al*. 2021*
	Limited guidelines for collaboration	*Hierarchical barriers , limited involvement of non-scientific community*	*Asaaga *et al*. 2021*
	Lack of inter-ministerial collaboration	*Lack of collaboration among ministries, limited focus on the veterinary sector*	*Asaaga *et al*. 2021, Dasgupta *et al*. 2021, Mckenzie SJ *et al*. 2016, Chatterjee P 2016, Taaffe J *et al*. 2023, Yasobant S 2019*
	Lack of coordinated OH research programs	*Lack of policies and frameworks, unutilized opportunities, limited guidelines, Lack of tools to capture field data*	*Bhat P *et al*. 2021, Tan Y *et al*. 2022, Prejit *et al*. 2022, Zhang X *et al*. 2023, Yasobant S 2019*
	Challenges faced by Veterinarians	*Vets have limited involvement in health-policy deliberation*	*Asaaga *et al*. 2021, Asokan VG *et al*. 2013*
	Challenges in practicing Citizen Science	*Limited awareness about Citizen science among scientists, Limited funding for community-driven projects, Existing policy failed to mention the potential of citizen science*	*Namdeo S *et al*. 2021, Patil R *et al*. 2021, Tan R *et al*. 2022*
	Challenges in publishing in Open access journals	*Open access trend: a boon to predatory journals, Publication inequality, Lack of funding support to publish in OA journals, Increasing article processing charges*	*Chakravorty N *et al*. 2022*

**II. Opportunities in the implementation of OH in the realm of OS:**

**Figure 2.2.**
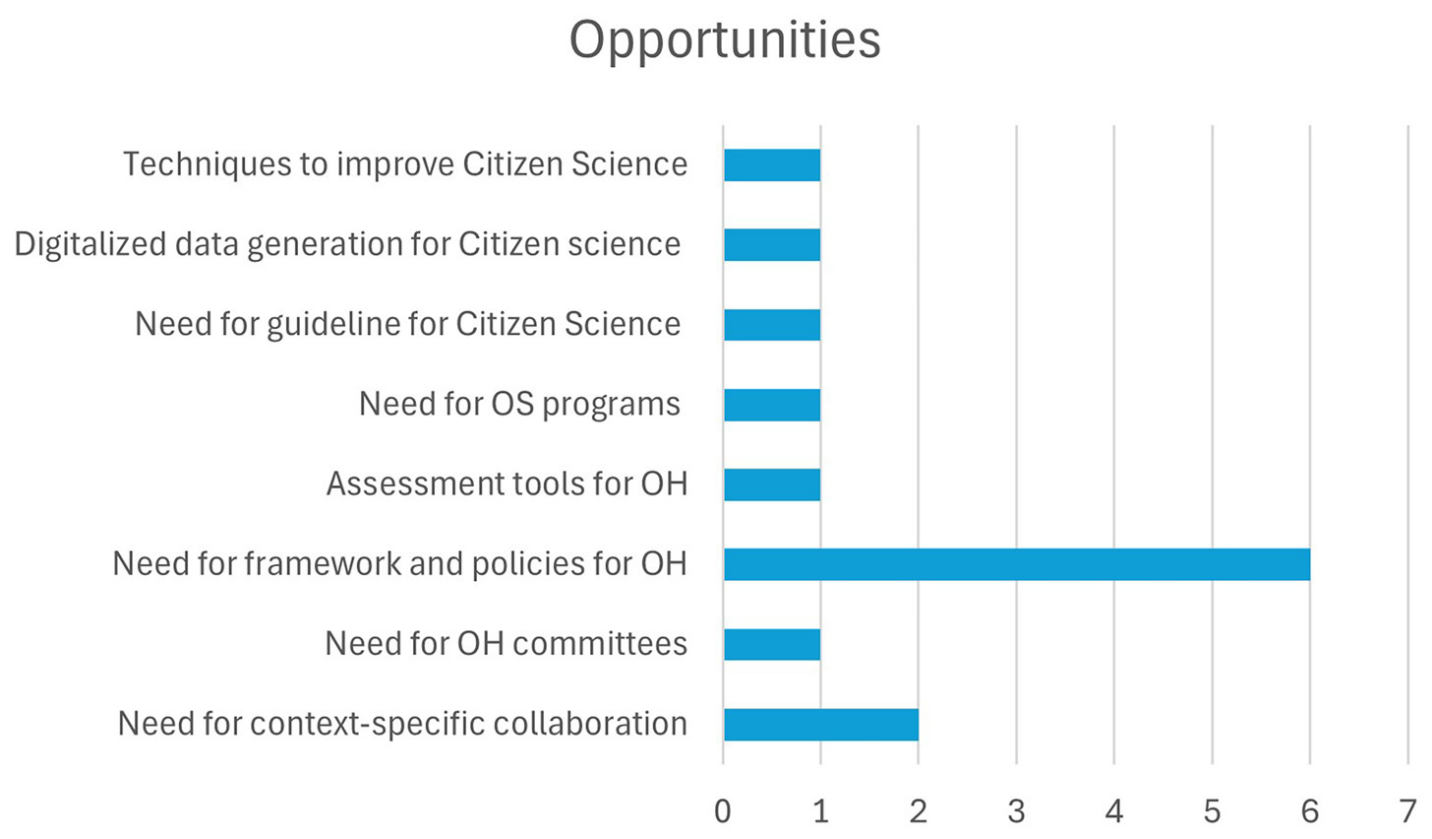
Distribution of Opportunities and the number of papers.

In addition to analyzing the challenges, the authors also identified four key opportunities for incorporating OS principles into OH. These opportunities, listed in Table 3.1 and Figure 2.2, provide strategies to overcome the existing barriers and foster collaboration, transparency, and data-sharing within the OH community in India.

**Table 3.1.** Opportunities to incorporate Open Science into One Health.

	Codes	Authors
**Opportunities**	*Need for One health committees*	*Dasgupta R *et al*. 2021*
	*Need for context-specific cross sectoral collaboration; facilitators*	*Asaaga *et al*. 2021, Yasobant S *et al*. 2020*
	*Need for Open science programs*	*Chakravorty N *et al*. 2022*
	*Assessment tools for One health*	*Prejit *et al*. 2022*
	***One health centric guidelines developed for each country***	*Abbas 2022, Yasobant S *et al*. 2019*
	*Need for framework and policies for One Health*	*Abbas S S *et al*. 2022, Bhat P *et al*. 2021, Chatterjee P *et al*. 2017, Yasobant S *et al*. 2021, Asaaga, *et al*. 2021, Narain JP 2016*
	***Techniques to improve Citizen science***	*Namdeo S *et al*. 2021*
	*Need for joint education for professionals*	*Asaaga *et al*. 2021*
	*Need for guideline for Citizen science*	*Namdeo S *et al*. 2021*
	*Need for equitable participation of stakeholders*	*Tan R *et al*. 2022*
	*Digitalized participatory data generation for Citizen science*	*Tan R *et al*. 2022*
	***Data visualization- to make results easily understandable***	*Tan R *et al*. 2022, Ulahannan JP *et al*. 2020*

## Challenges at the intersection of OH into OS in India

### a. Challenges in the implementation of OS into OH at Institutional level

The current infrastructure and support for the OH initiative in India face several challenges, including communication issues, resource allocation problems, gaps in knowledge and learning ^14^. Challenges like limited infrastructure, funds, lack of zoonotic disease knowledge, inadequate training, limited response capacity, political conflicts between different sectors, and unclear reporting systems for human and animal diseases contribute to the difficulties in implementing OH in India at both local and central levels ^15^. The same challenges also become significant limitations in practising OS within OH. Open Science depends on strong infrastructure, transparent data sharing, clear communication channels, and active collaboration across disciplines. When infrastructure is weak and funding is limited, the development of open-access platforms, databases, and communication systems becomes extremely difficult.

Moreover, limited zoonotic disease knowledge and inadequate training mean that researchers may lack the skills and capacity to collect, manage, and share high-quality data openly. Political conflicts and unclear reporting systems restrict the free flow of information that is essential for OS to thrive. To address these challenges, it is crucial to promote interdisciplinary collaboration, make infrastructural changes, invest in advanced technology and policymaking ^16^. This includes establishing interdisciplinary laboratories that facilitate problem-solving and strengthening collaborations between research institutions like ICMR (Indian Council of Medical Research)-ICAR (Indian Council of Agricultural Research)/RCZI (Road Map to Combat Zoonoses). This in turn shall be achieved through increased funding, staffing, resources, and political support ^17^.

### b. Challenges in the implementation of OS into One Health at National level

**i. Barriers to cross-sectoral collaborations**

According to Abbas *et al*. ^18^, the concept of multisectorality in the OH approach consists of different agendas aligned towards a common goal. The definition of sectors can vary, from different disciplines or institutions ^18^. During the 12 ^th^ five-year plan, the Department of Health and Family Welfare, India launched a scheme to strengthen inter-sectoral coordination for the prevention and control of zoonotic diseases in 2012. The objectives of this program included establishing coordination, communication, laboratory, capacity building, and awareness for effective prevention and control ^19^.

However, Mckenzie *et al.* ^20^ in their critical review mentions that without effective surveillance mechanisms and information sharing between sectors, forming a complete picture becomes challenging. Although multisectoral committees have been established, collaborative research and policy formulation are yet to be implemented effectively. The authors suggested that joint education and training of professionals from various sectors can help build understanding, trust, and break down barriers between different disciplines ^20^.

Besides, a mixed-method study by Asaaga *et al.* ^21^, revealed several barriers to cross-sectoral collaborations such as communication and information gaps, differences in disciplinary training, and institutional challenges like bureaucratic hurdles, perceived mistrust, ego clashes, differing mindsets, insufficient funding, and coordination issues. Additionally, contextual factors such as hierarchies and regional capacity disparities were also seen as constraints. The authors stressed the importance of effective communication between all sectors and levels for promoting cross-sector engagement. According to Asaaga *et al.* ^21^, the hierarchical structure of the health system often gives national-level agencies significant powers, potentially weakening support for local health priorities. This can lead to a shift in focus from local priorities to those dictated by central government, particularly in cases where state-level health departments lack adequate funding. The rigid hierarchy leads to weak self-feedback mechanisms within the health system, inhibiting effective communication and feedback from ground-level workers to their superiors ^21^.

State government agencies encounter human resource and logistical challenges, with varying administrative cultures across different states in India. The disparities in administrative practices and the lack of skilled personnel slow down collaborative actions. This makes the officials transfer the tasks to other authorities ^21^. Participants in the study conducted by Asaaga *et al.* ^21^ also expressed a lack of willingness to engage in collaborative actions across sectors due to time constraints and increased administrative burden. They emphasized the need to sensitize all actors about the importance and benefits of such collaborations. State and district level actors reported feeling overwhelmed by competing demands, making it difficult to balance their responsibilities.

During outbreak events, veterinarians and medical professionals collaborate and develop mutual respect and trust. Short-term collaborations during outbreaks are more likely than long-term systematic collaborations, as the latter requires formalized understanding between different institutional bureaucracies ^21^. Collaboration is only emphasized when deficiencies are found within the respective sector or when services are required from other sectors. Top-down directives drive collaborations during outbreaks, leading to power issues in non-outbreak situations. Additionally, collaborations are perceived as burdens rather than benefits, leading to further challenges ^22^.

There is also a discrepancy in shared responsibilities between central and state governments, making inter-sectoral merging difficult ^23^. Time constraints and additional administrative burdens further affect collaboration, with state and district-level actors feeling overwhelmed especially during disease outbreaks. Balancing clinical and administrative responsibilities is particularly challenging during such crises, impacting their capacity to engage in cross-sectoral efforts, which is a key aspect of OS.

***Challenges in data sharing***

When researchers open up their data, they make it possible for others to check their work, reproduce their results, and build new ideas to what is already known. This kind of openness breaks down barriers between disciplines and sectors, helping people solve problems together. It may also save time and resources by reducing the need for researchers to collect the same data repeatedly. In 2019, Bhat *et al.* ^24^ conducted a mixed-method study to evaluate the existing surveillance system for Kayasanur Forest Disease (KFD) in Shivamogga, Karnataka, India. According to the authors, the global policy on the OH approach focuses on combining the health of humans, animals, and the environment. This requires collaboration, cooperation, and data sharing among stakeholders and different departments ^24, 25^. The KFD study revealed that the existing surveillance system had questionable practices regarding timeliness and quality of reporting data. The authors stress about the importance of reporting data that is easily understandable to all. There is also a great need to establish a robust data sharing mechanism between animal husbandry and forest department staff to enhance the comprehensiveness of the surveillance system ^24^. For scientists in low-resource settings, access to open data can be a lifeline, giving them tools they couldn’t otherwise afford. Therefore, design and development of simple surveillance tools based on stakeholder feedback would be helpful to effectively utilize field data, capture quality data, and take appropriate actions ^26, 27^.

Dasgupta *et al.* ^28^ in their review article in 2021, expressed the need to visualize the OH concept beyond the scope of zoonotic diseases. According to the authors, the challenges in OH issues in South Asian countries point towards the need for productive engagement between medical, laboratory, and social scientists. While OH has been institutionalized in some South Asian countries, further intersectoral institutional changes are needed to strengthen OH research and training ^28^. Globally, there are several OH initiatives in progress, but the Indian approach has primarily been focused on developing solutions during disease outbreaks, rather than fostering sustained, proactive research and preparedness ^28^. Integrating OS principles such as open data, collaborative platforms, and transparent sharing of research findings into India’s OH approach could help build a more resilient system. By encouraging ongoing collaboration between human, animal, and environmental health sectors, OS can shift the focus from crisis response to continuous innovation and prevention.

According to Dahal *et al.* ^29^, Bangladesh and India have made progress towards OH approach but struggle to bring the governmental sector and stakeholders together for joint action. This could be due to organizational structure, staff attitudes, or cultural and perception issues that require behavioural changes among professionals ^28, 29^. Barriers to sustaining cross-sectoral action include mistrust, differences in mindsets, and ego clashes. Public health experts often view themselves as primary leaders in cross-sectoral collaborations, while experts in the animal health and environment sectors perceive that the public health experts take charge of recognitions from cross-sectoral efforts. There is also a belief that each sector has its own mandate and does not necessarily have to implement policies or guidelines from other sectors ^21, 30, 31^. According to Sironi *et al.* ^32^, the OH approaches may be challenging for many countries, as the new framework requires greater investment and can be more expensive to implement. In summary, there is a need for improved collaboration and understanding among sectors in South Asian countries to effectively address OH issues. This requires overcoming cultural and perception issues, behavioral changes, and better intersectoral communication and engagement. Moreover, when the public has funded the research, sharing the data is a way to give back to society, ensuring that knowledge serves everyone, and not just a few.

***Limited guidelines for collaboration***

According to Asaaga *et al.* ^21^, there were limited guidelines on fostering, managing, and sustaining cross-sectoral collaborations. Key sectoral policies lacked clear formal guidelines or frameworks to support cross-sectoral action, which constrained sustained coordination efforts outside of outbreaks. The authors emphasized a great need for operationalization guidelines specifying roles in disease control efforts across relevant departments. Without structured guidelines, the OH actors felt collaboration was not possible, and they could not visualize collaboration during non-outbreak periods.

However, most collaborations were based on specific instructions from top authorities during outbreaks ^22^. The study by Asaaga *et al*. also highlights that implicit hierarchies exist within the social setting and professional relationships, particularly in contexts where career sensitivities are prominent. These barriers to cross-sectoral collaboration extend beyond technical aspects and are deeply rooted in the dynamics of collaboration. Creating an environment where professionals from different disciplines come together and have mutual respect is a significant challenge. One potential solution could be incorporating transdisciplinary modules in the curriculum to expose students to the value of interdisciplinarity. The authors also stressed on the need for context-specific cross-sectoral collaboration ^21^.

Dasgupta *et al.* ^28^, argue that the absence of a policy framework that officially recognizes the OH approach in development and health policies hinders efforts to eradicate poverty and poverty-related diseases ^28^. Prejith *et al.* ^14^ note that there are still no validated guidelines for quantitatively measuring OH activities ^14^. Mckenzie *et al.* ^20^ express a significant need for well-coordinated and well-designed research programs on zoonotic diseases within a OH framework to develop effective integrated disease control policies for both humans and animals ^20^. Limited cross-sector engagement exists for most zoonotic diseases due to the lack of an integrated OH policy. Effective implementation of OH requires a framework that includes policy formation, program implementation, financial support, interdepartmental collaboration, capacity building, and local-level participation ^21, 33^.

However, the actors involved in cross-sectoral collaboration range from administrative policymakers and program implementers at the highest level to community actors such as village leaders and health workers at the lowest level. To facilitate collaboration, Raut *et al.* ^23^ in their narrative review suggest robust methodological tools like transdisciplinary multicriteria analysis and a health management system thinking approach have been proposed. Additionally, the Checklist for Reporting of OH Epidemiological Evidence (COHERE) has been employed to combine knowledge from the animal, human, and environmental sectors ^23^.

***Lack of inter-ministerial collaboration***

One major challenge in implementing OH initiatives is the difficulty in achieving inter-ministerial collaboration to establish a common funding strategy ^28^. The participants in the study by Asaaga *et al.* ^21^ stated that fragmentation and disparate sectoral affiliations make it difficult to achieve cross-sectoral collaborations in India, as different ministries and departments have different goals and power ^21, 34^. Besides, there is a lack of comprehensive guidelines on coordinating between sectors and departments, leading to political decision-making rather than technical decision-making. The nature of government agencies in different disciplines hamper cross-sectoral collaboration. Each department believes that all expertise lies within itself, further contributing to the challenge ^21^, which poses a major obstacle to promote OS.

Additionally, the compartmentalization of the human health and animal health departments under different ministries contributes to bureaucratic competition and affect the implementation of cross-sector initiatives. While formal collaborative mechanisms were established in South Asian countries to address emerging disease threats, the sustainability of these collaborations for managing endemic zoonoses during non-outbreak periods has been inconsistent. Some countries have made progress in building a strong OH network and institutionalizing OH, while others rely on individual advocates and donor funding for collaboration. Bangladesh stands out as a leading example, having signed the OH Strategic Framework in 2012 and making consistent progress in implementation ^20^. India also initiated multisectoral collaboration in response to the H5N1 influenza outbreak and formalized it through an Inter-Ministerial Task Force and Joint Monitoring Group ^35^. However, coordination among ministries such as Health, Agriculture, Wildlife, and Environment remains challenging due to differing objectives and priorities.

To enhance the Indian health system, robust and integrated OH and OS initiatives beyond outbreaks are required. The Department of Biotechnology under the Ministry of Science and Technology has taken steps toward establishing an inter-ministerial OH task force, which could help bring essential ministries under its umbrella. However, institutional and administrative problems continue to hinder collaboration across ministries, making the operationalization of OH challenging ^17^.

***Lack of interdisciplinary collaboration***

According to Asaaga *et al.* ^21^, the wildlife sector has had limited involvement in health policy deliberations compared to the human health and veterinary sectors in India. This lack of representation from the wildlife sector is evident in committees such as the National Standing Committee on Zoonoses. Besides, there is a longstanding perception among veterinarians that they are considered less prestigious compared to human doctors, which creates a competitive dynamic between the two professions. However, there is potential for collaboration between veterinarians and doctors during disease outbreaks, as illustrated by their cooperation in cases of dog bites and rabies ^21^. In fact, Veterinary institutes are essential for OH research and diagnostics. During the Covid-19 pandemic, they demonstrated their capability in supporting human disease outbreaks by providing diagnostics, screening, and logistical support ^15^. Therefore, there is a need to strengthen the health care system by promoting knowledge translation, exchange, and other activities between the veterinary and medical health sectors ^36^.

***Lack of coordinated One health research programmes***

According to Mckenzie *et al.* ^20^, several significant initiatives have been undertaken in India to address zoonotic diseases. This includes the establishment of a National Standing Committee on Zoonoses in 2005, responsible for advising on policies, operational research, and inter-sectoral collaboration for zoonotic disease control. Additionally, in 2010, the U.S. Centers for Disease Control and Prevention (CDC) and the National Center for Disease Control in India together agreed to setup the India Global Disease Detection Regional Center. Nevertheless, this partnership aimed to enhance epidemic intelligence services, surveillance, preparedness and response to outbreaks, laboratory safety, and zoonotic disease investigation and control. Despite these efforts addressing potential pandemic threats, they have yet to fully institutionalize a OH approach supporting coordinated research programs and effective integrated policies for controlling zoonotic diseases ^20^.

However, the emergence of avian influenza H5N1 in the year 2006 ^37^ helped highlight the need for multisectoral collaborations in India between human health, animal health, and wildlife sectors. Institutionalized efforts included the establishment of an Inter-Ministerial Task Force and Joint Monitoring Group at the national level, with coordination mechanisms extending down to the district level. To address these challenges, a national-level OH Consortium was established by the Department of Biotechnology - National Institute of Animal Biotechnology. This comprises 27 organizations including medical, veterinary, and wildlife sectors. Additionally, efforts towards establishing a National Institute of One Health in India have received joint support from the Indian Council of Medical Research (ICMR) and the Indian Council of Agricultural Research (ICAR) ^21^.

 Although, the opportunity created by these initiatives has not been fully utilized, and there is a need to expand their scope to address zoonoses and other issues at the human-animal-wildlife interface ^21^. OS, with its emphasis on transparency, data-sharing, and interdisciplinary cooperation, aligns well with these objectives, offering a pathway to strengthen OH implementation.

### Ii. Challenges in practicing Citizen science

Citizen Science plays a vital role in advancing OS by involving the public in scientific research, data collection, and knowledge production. By bridging the gap between professional scientists and communities, Citizen Science democratizes science, enhances transparency, and fosters wider access to scientific processes and outcomes. In a diverse country like India, the potential for Citizen Science to contribute to sustainable development, informed policy-making, and community empowerment is immense. However, as Namdeo *et al.* ^38^ highlights, there is a shortage of Citizen Science projects, publications, and participants in India when compared to the country's capacity. Current volunteers mostly come from the educated middle class, representing only a small portion of the population. Addressing the challenges and opportunities associated with Citizen Science in India is the need of the hour. With South India having the highest number of projects, uneven geographic and thematic distribution of projects is a major issue ^38^. Secondly, the data quality from Citizen Science projects in India is often questionable due to personal biases and lack of trained volunteers, leading to concerns among researchers.

As a result, there is mistrust among scientists regarding data collected by volunteers or communities. Financial stability, particularly for community-driven projects, is also a concern ^26, 38^. Nevertheless, issues of data ownership, usage, privacy, and accessibility are also the major challenges in the Citizen Science approach ^38^. Kullenberg and Kasperowski ^39^ in their scientometric meta-analysis located public participation in epidemiological research under the Citizen Science umbrella ^39^. In a scoping review by Hansen *et al.* in Denmark ^40^, the challenges in the research data management by citizen science is enormous, such as- difficulties in the identification of the data of citizen science origin, sharing of data by the citizen scientists before the publication of the findings, and challenges in reusability of data.

The authors also stress upon lack of databases and publishing platforms for citizen science. In addition, the authors state that most of the citizen scientists are considered “Research Assistants” and not research participants, due to which the Institutional Review Boards (IRBs) do not assess the risks and benefits of the citizens. In our study, we explored a group of community health workers in India called Accredited Social Health Activists (ASHAs). They act as a bridge between the community and the health service providers ^39^. There are about 9,00,000 ASHAs in rural and 64,000 in urban areas working under the National Health Mission (NHM), India ^41^. According to Ghanekar *et al*. ^41^, there are several gaps in research on Community Health Worker (CHW) programs, and the published literature often fails to discuss essential health system considerations, such as program governance, community voice and engagement, and collaboration between the community and CHWs. According to Rajbangshi *et al.* ^42^, displacement of ASHAs in conflict-hit areas has significantly affected the living conditions and perception of security risks among ASHAs ^42^. Disparities in social access between urban and rural ASHAs have also been observed, emphasizing the need for greater social inclusion in urban ASHA programs ^41^.

They are extensively involved in various public health programs and interventions, demonstrating their multidisciplinary working culture ^43^. They also collaborate with schoolteachers and participate in mass sanitation campaigns. However, the incentive-based payment system poses a significant challenge, as workers may not receive financial incentives due to non-completion of tasks or unavailability of data. Additionally, there is dissatisfaction with the lack of fixed work schedules and disparities in incentive distribution among workers. Despite their extensive efforts, there is a lack of equal compensation, particularly during outbreaks, which impacts their motivation and financial stability ^44– 46^. There is a need for formal policies to address these challenges, as well as the provision of incentives and recognition ^14^.

Additionally, factors such as training, supervision, and security are crucial for engaging community health workers in research. In a case study by Yasobant *et al.* ^46^ in 2021, the ASHAs expressed a strong desire to represent themselves as OH activists, if relevant incentives and recognitions are given to them. They also expressed a need for proper training in the field of OH. Proper training and sensitization of these multipurpose healthcare workers could enhance their ability to work on zoonoses prevention effectively ^22^.

### Iii. Challenges in publishing in open access journals

Chakravorty *et al.* ^47^ in their article of 2022, discussed the challenges in the implementation of OS, and various OS initiatives of India. The first and the foremost challenge is the cost of journal subscriptions increasing significantly over the years, even with the availability of online access. According to the authors, this is a concern because it creates barriers for universities and institutes worldwide. Research publishing giants like Elsevier, Wiley-Blackwell, Springer, and Taylor & Francis dominate the market, publishing more than 50% of global research products. Besides, developing countries often do not receive waivers for publication costs, despite being classified as low-income or lower-middle-income economies. Secondly, many journals are unwilling to publish negative results, which leads to publication biases and a lack of transparency in research ^47^.

According to Misra *et al.* ^48^, predatory journals take advantage of the open access model by publishing low-quality articles for a fee without proper peer review. They often publish articles rapidly and without sufficient review, leading to the publication of scientifically inappropriate content. Researchers can unknowingly fall victim to these predatory publishers, compromising the quality and reliability of their work. Therefore, the authors suggest strict measures to ensure rigorous peer review ^47^. Sinclair ^49^ in his review of a commentary in 2019, stressed the implementation of OH as the one-stop solution for the rise in zoonotic diseases and anti-microbial resistance (AMR). Nevertheless, the OH framework can improve global health by collaborating with all the stakeholders who work at the human-animal-environment interface ^6^.

On the other hand, Reichmann and Weiser ^50^ recommend OS to enhance scientific developments, as it helps the policy-makers utilize the publicly available research results and data. Furthermore, the practice of OS in health research promotes increased transparency in data collection and methods, encouraging reproducibility and accuracy ^51^. Therefore, there is a need to explore the ethical issues in the practice of OS in OH, thereby engage, equip and guide the OH community in the application of the principles of OS and promote ethics and research integrity in OH.

## I. Opportunities in the implementation of OH in the realm of OS

### One Health centric guidelines developed for each country

The integration of OS principles into OH initiatives present significant opportunities for overcoming the existing challenges in India. According to Yasobant *et al.* ^17^, challenges such as lack of awareness, access, human resource crisis, affordability, and lack of accountability in OH can be overcome by sustainable collaboration and open-knowledge sharing frameworks ^17, 52^. Besides, multilateral agencies have developed specific OH-centric guidance to facilitate country-level OH collaborations ^18^. These global One Health Collaboration (OHC) initiatives are considered as potential approaches for the Indian context.

In addition, the establishment of the National Institute of OH has been a major development ^53^. However, the lack of documented evaluation makes it difficult to speculate which of these initiatives would be most effective in developing a resilient Indian health system capable of adapting the OH approach. Therefore, the authors express the need to develop specific local strategies for the Indian context ^17^. This underscores the need for localized OS strategies, including community-driven data-sharing platforms, open-policy dialogues, and participatory research initiatives that address India's specific challenges in implementing OH.

### Existing Open science programs in India

Chakravorty *et al.* ^47^ in their review article of 2022, listed several initiatives in India that are aimed to promote OS and improve access to scientific resources. The Indian Academy of Sciences and the Indian National Science Academy publish journals and provide online access to them. The National Digital Library of India offers a virtual repository of learning tools and has a vast collection of resources. The Vigyan Prasar Digital Library serves as a repository of digitalized scientific works.

Shodhganga, maintained by the University Grants Commission, provides public access to a central repository of theses and dissertations. Open Access India (OAI) advocates for open access, open data, and open education in the country and played a key role in the formulation and promotion of the Delhi Declaration, which includes ten points advocating for various OS practices. The Department of Science and Technology, Government of India, has expressed its intent to establish an OS Framework through the fifth Science, Technology, and Innovation (STI) policy draft, which aims to bring fundamental changes in the field of science and promote research and innovation. Additionally, the draft policy mentions the creation of a National STI Observatory as a central repository for data and emphasizes the promotion of inclusivity in science education at all levels. These efforts demonstrate a commitment to advancing OS in India ^47^. These efforts contribute to making science more accessible in India.

### Techniques to improve Citizen Science

As technology becomes more prevalent, digital-based systems for data generation are rapidly advancing. According to Chakravorty *et al.* ^47^, several analytical techniques have been developed to filter data for quality due to personal biases. These techniques include iterative project development, volunteer training and testing, replication across volunteers, expert validation, and statistical modeling of systematic errors. These strategies are aimed at improving data accuracy ^47^. The Covid-19 pandemic has also increased the adoption of digital technologies in healthcare settings, which can address the limitations of traditional participatory approaches, and moving towards digitalized participatory data generation for Citizen Science ^26, 54^.

### Data visualization- to make results easily understandable

Tan *et al.* ^26^ in their narrative review, highlighted the importance of tailoring data visualizations for easy understanding of the data by different stakeholders. Citizen science approaches involving the community have been used to design visualizations, empowering villagers to make informed decisions through data as seen in the ``Participatory Tracking Project`` conducted in Tamil Nadu. The second example is of the visualization dashboard developed for the COVID-19 outbreak in Kerala, which has a bilingual interface and unique features. These initiatives demonstrate the potential for efficient data management and the use of community-led initiatives in citizen science in India ^26, 55^. To conclude, data science is pivotal in advancing healthcare, offering numerous applications and benefits, with future prospects that could significantly enhance healthcare delivery systems ^56^. However, there is a need to ensure data privacy which indicates the importance of having related regulations in India ^57^.

## Discussion

This article intended to map the literature on the challenges and opportunities in OH in the era of OS in India. The scoping review identified 46 articles that addressed topics relevant to implementation of OH showing that inter-sectoral collaboration, field data storage, and ASHA workers remain the prevalent challenges of OH researchers in practicing OS. It is important to note that ethical issues embedded in OH were not specifically mentioned in the articles. However, this can be explained by the nascent stage of OH implementation in India where challenges are at pragmatic level.

At the institutional level, barriers to *cross-sectoral collaborations* were identified as a significant challenge ^21^. This suggests that there is a lack of coordination and cooperation between different sectors, such as the medical and veterinary sectors, which hampers the implementation of OH. Cultural challenges were also identified, indicating that there may be differences in attitudes and practices between different stakeholders that need to be addressed ^21, 23^. Furthermore, the lack of inter-ministerial collaboration suggests that there is a need for better coordination and communication among government agencies involved in OH ^15, 17, 21, 28, 58^.

Another challenge at the institutional level is lack of interdisciplinary collaboration. This highlights the need for greater recognition and involvement of veterinary professionals in health-policy deliberations ^21, 36^. Additionally, hierarchical barriers between veterinarians and medical professionals were identified, which could hinder effective collaboration and information sharing ^21^. Collaboration helps bridge gaps in resources, funding, and technological infrastructure, fostering a more equitable global scientific landscape. By uniting scientists with non-academic stakeholders, OS becomes more effective in tackling global issues ^29^. Our findings corroborate earlier study’s finding that although solution-based OH initiatives or activities in India during outbreaks or health emergencies are robust and implemented at the grassroots level, there is little incentive for intersectoral collaboration. Outside of outbreaks, collaborations tend to be limited to specific levels or dependent on third party partnerships, rather than being fully integrated across sectors ^15^. Without collaboration with policymakers, industry, and society, researchers may struggle to address real-world challenges.

Studies involving human participants, indigenous knowledge, or sensitive data require diverse perspectives to uphold ethical standards. Therefore, a lack of collaboration can lead to exploitation or harm to vulnerable populations. Nevertheless, cross-sectoral collaboration enhances transparency in research methodologies, data sharing, and findings. UNESCO ^1^ encourages open access to scientific data to promote reproducibility and credibility. It also underscores the importance of inclusivity in OS by motivating active participation from underrepresented groups, such as researchers from developing countries, indigenous communities, and citizen scientists. Similarly, the Responsible Open Science in Europe (ROSiE) guidelines ^59^ advocate for responsible OS and inclusivity, ensuring that ethical considerations and societal needs are fully integrated into research. It stresses upon the need to engage diverse communities to promote inclusivity and uphold ethical standards.

Data sharing was another significant challenge identified in this study. The absence of clear data sharing guidelines raises concerns about privacy, ownership, and the potential exploitation of local or indigenous knowledge. As a result, the lack of data sharing in OH research, along with confidentiality issues, suggests that there is a need for improved mechanisms and tools for capturing and sharing field data ^24, 26^. At the national level, challenges related to Citizen Science were identified ^26, 38^. Limited awareness about Citizen Science among scientists and limited funding for community-driven projects indicate the need for greater promotion and support for Citizen Science initiatives. Additionally, the existing policy framework failed to mention the potential of Citizen Science, highlighting the need for policy reforms to incorporate and support such initiatives ^14, 16, 20, 21, 28, 33^.

Moreover, in a low and middle-income country like India where funding opportunities for research is scarce, practicing OS may lead to several inequities and exploitation. For example, no longer having access to shared information poses one of the major obstacles to practicing OS. Without accessible data-sharing platforms, research findings remain isolated, hindering collaboration and slowing scientific research. This lack of openness contradicts principles of equity and inclusivity. When researchers cannot access existing data, studies may be unnecessarily duplicated, leading to wasted resources, time, and effort. Scientific integrity relies on verifiability, but without proper data-sharing mechanisms, research cannot be independently validated, increasing the risk of errors, bias, and potential misconduct.

Researchers from low-resource institutions or developing countries often face barriers to valuable datasets, exacerbating global disparities in scientific advancement. Limited data sharing can also delay progress in OH research, impeding timely responses to public health crises. Citizen Science is an integral part of OS, as citizens are often the best individuals to identify the challenges their communities face. For instance, during a disease outbreak, the community experiences its impact first, making them the best contributors in selecting research topics and designing appropriate study designs. This approach is central to Community-Based Participatory Research (CBPR). When communities actively participate in the research process, it becomes the responsibility of the researcher to share the study findings with them. These results should be communicated in an easily understandable manner, ensuring that community members can benefit from the knowledge they contributed to. Denying them access to this data would violate the principle of benefit-sharing in research.

To resolve these ethical concerns, it is essential to develop inclusive, secure, and FAIR data-sharing platforms that promote responsible research while upholding privacy, equity, and transparency. The FAIR data principles offer a strong framework for ethical data sharing. Ensuring that data adheres to these principles enhances transparency, fosters global collaboration, and supports reproducibility ^59^. Without this, research is obstructed, and issues like data hoarding and the exclusion of underrepresented communities persist. Implementing FAIR guidelines is key to ensuring ethical, responsible, and open research practices. At the same time, health research often involves sensitive data and that must balance the FAIR principles with the ethical responsibility to protect personal and community privacy. This creates an ongoing tension: while open and FAIR data are crucial for rapid response, knowledge sharing, and collaborative problem-solving, they must not compromise individual privacy, and other ethical standards. Without strong governance frameworks, secure data management platforms, and clear policies on consent and data anonymization, the push for open data can clash with the duty to safeguard personal information. This further complicates the efforts to practice true OS in OH. Thus, addressing this challenge is as important as addressing infrastructure, capacity, and collaboration gaps to make both OS in OH effective and trustworthy.

Other challenges identified include issues related to *publication bias* and the *increasing article processing charges* ^47^. Furthermore, the lack of funding support for publishing in Open Access journals highlights the need for greater financial support for researchers in this area ^15, 47, 60^. However, there is limited literature available on the practice of OS principles in India. This shows that there is lack of awareness about OS principles, tools and techniques in India. Addressing these challenges will contribute to the advancement of OH in the improvement of health outcomes for human, animal and the environment in India, and other LMICs.

This study also explored several opportunities to incorporate OS into the OH framework, identifying various initiatives and areas for improvement. We looked at existing OS initiatives in India mentioned by Chakravorty *et al.* ^47^, which provide a valuable model for how transparency and accessibility in research can be improved. In terms of areas for improvement, several key recommendations were identified which are crucial for the successful implementation of OH. First, there is a need to *establish OH committees*, a great initiative highlighted by Dasgupta *et al.* ^28^. Moreover, Asaaga *et al.* ^21^ rightly emphasized the necessity of *context-specific cross-sectoral collaboration* to address the unique challenges of different regions effectively. Furthermore, Namdeo *et al.* ^38^ underscored the importance of *developing guidelines* for citizen science, which would standardize practices and enhance the quality and reliability of data collected by the public. Finally, Tan *et al.* ^26^ highlighted the need for *equitable participation of all stakeholders*, ensuring that every voice is heard and considered in decision-making processes. According to Tulchinsky ^61^, one of the ethical principles of public health is transparency, therefore, the strategies adopted to ensure honesty and trustworthiness should be transparent and reliable.

## Conclusion

Overall, the results of this review demonstrate that the implementation of OH in the context of OS is faced with several challenges at both the institutional and national levels. The issues identified were lack of inter-ministerial collaboration, hierarchical barriers, limited attention to the veterinary sector, challenges in data sharing, and challenges at the level of community health workers. Therefore, it is important to address these issues to ensure effective collaboration and responsible conduct of research. To support its long-term success, it's crucial to develop context-specific OH policies in the backdrop of OS principles. These policies should emphasize openness of data sharing, foster cross-sector collaboration, and digital infrastructure. Cross-sector collaboration brings together academia, industry, government, and society and helps in realizing the full potential of OS. Moreover, inclusive participation and responsible research practices are also critical to ensure that OS is not only transparent and ethical but also beneficial to society. Therefore, embedding OS within OH governance can lead to a more transparent, inclusive, and responsive public health system, that is better equipped to tackle complex challenges at the intersection of human, animal, and environmental health.

## Limitations

The review was limited to English-language publications, which may have excluded relevant regional literature. Additionally, ethical dimensions in the reviewed studies were often implicit rather than explicitly discussed, requiring inferred analysis. The scope was restricted to India, limiting broader regional comparisons. Furthermore, our analysis was a-theoretical, as we did not apply a formal framework to guide data analysis or theme categorization. The initial keyword search was conducted on PubMed and Web of Science, but finding articles relevant to OS and OH in the Indian context proved challenging. To address this, we employed multiple keywords. While searches on Embase, Medline, and Global Health were conducted with the assistance of a librarian, the choice of keywords remained limited to maintain focus on the study’s objectives. Consequently, we included only articles that explicitly discussed challenges in OH, data sharing, ethics, OS, and Citizen Science. Since both OH and OS are still emerging fields in India, we also incorporated a few global studies that addressed OH challenges in low- and middle-income countries (LMICs). These were included because India falls within this category, and their insights were considered relevant to our study's context.

## Future directions

This study primarily focused on the practice of OS within the Indian context. However, further research should expand to include a global perspective. Comparing the challenges and impacts of OS between the Global South and the Global North will provide a more comprehensive understanding of the global disparities and inequities in OH research. Additionally, future studies should explore the perceptions of OH stakeholders regarding the inequities that OS practices may exacerbate or mitigate. Such qualitative studies will help explore the nuanced socio-economic, cultural, and infrastructural factors that influence equitable participation in OS initiatives. In the next phase of our project, we aim to explore how these inequities manifest in the South Asian OH scenario. This will involve engaging diverse stakeholders to identify barriers and propose solutions for fostering a more inclusive and equitable OS framework. This could inform policies and practices to ensure that OS fulfills its potential to democratize knowledge and promote global health equity.

## Ethics and consent

The study was approved by the Yenepoya Ethics Committee-2 (YEC2/2023/044), India. Consent was not required for the study.

## Data Availability

The data used in this article comprises of bibliographic references, which are listed in the References section. The dataset is available in the Zenodo repository. Zenodo: Ethical Challenges and Opportunities at the intersection of One Health and Open Science in India: A Scoping Review. https://doi.org/10.5281/zenodo.15860636 ^62^. - Dataset scoping review: PRISMA-ScR-Fillable-Checklist.pdf Search strategy and articles retrieved.docx ScR NViVO.nvp Codebook-ScR.pdf Extended data: https://doi.org/10.5281/zenodo.17159506 ^63^ Data are available under the terms of the Creative Commons Attribution 4.0 International license (CC-BY 4.0). Statement on the use of Artificial Intelligence (AI): The authors confirm that no AI tools were used in the preparation of this manuscript.
