# Supplementary material for: Ethical Challenges and Opportunities at the Intersection of One Health and Open Science in India: A Scoping Review
Source: Open Res Eur. 2026 Jan 27;5:302. Originally published 2025 Oct 6. [Version 2] doi: 10.12688/openreseurope.21085.2 (PMC12759278; doi:10.12688/openreseurope.21085.2)
Supplement: Peer review history

# Reviewer report: Reviewer response for version 2

**DOI:** 10.21956/openreseurope.24773.r68692

**Published:** 2026-02-05

**Authors:** Joseph Mwanzia Nguta

**Affiliations:** 1 University of Nairobi, Nairobi, Nairobi, Kenya

Approved. No further comments to make.

Are the rationale for, and objectives of, the Systematic Review clearly stated?

Yes

Is the statistical analysis and its interpretation appropriate?

Yes

Are sufficient details of the methods and analysis provided to allow replication by others?

Yes

Are the conclusions drawn adequately supported by the results presented in the review?

Yes

Reviewer Expertise:

One Health research ethics; One Health research; Ethics of One Health research

I confirm that I have read this submission and believe that I have an appropriate level of expertise to confirm that it is of an acceptable scientific standard.

# Reviewer report: Reviewer response for version 2

**DOI:** 10.21956/openreseurope.24773.r68694

**Published:** 2026-02-04

**Authors:** Sara Savić

**Affiliations:** 1 Scientific Veterinary Institute, Novi Sad, Serbia

All the comments and suggestions that were placed by myself, were addressed and commented on. The authors have improved the manuscript according to the suggestions.  I have no further comments and my opinion is that the paper is good to be indexed.

Are the rationale for, and objectives of, the Systematic Review clearly stated?

Yes

Is the statistical analysis and its interpretation appropriate?

Yes

Are sufficient details of the methods and analysis provided to allow replication by others?

Yes

Are the conclusions drawn adequately supported by the results presented in the review?

Yes

Reviewer Expertise:

NA

I confirm that I have read this submission and believe that I have an appropriate level of expertise to confirm that it is of an acceptable scientific standard.

# Reviewer report: Reviewer response for version 1

**DOI:** 10.21956/openreseurope.22807.r65783

**Published:** 2026-01-07

**Authors:** Sara Savić

**Affiliations:** 1 Scientific Veterinary Institute, Novi Sad, Serbia

The manuscript contains all the needed parts such as Introduction, Methodology, Results, Discission and Conclusion. The writing in English language is good and well used. The graphs and tables are created in use of the better understanding of the results.

 There are several points where manuscript can be improved:

 Page 4,  Title and abstract screening , the sentence  “This was performed by NH, AC and UK.” Is not easy to understand and should be “This was performed by the authors NH, …)  

 Page 5,  Data extraction and charting, the type of publication is not mentioned in Figure 1, so no need to mention Figure 1 in the following sentence “ We reviewed 33 original articles, 2 policy briefs, 2 commentaries,

 4 opinion pieces, 2 case studies, 2 protocols, and 1 case report (Figure 1.0).”

 Page 10, the paragraph starting with sentence “According to Dahal et al.29, Bangladesh and India have

 made progress towards OH approach…” is about OH challenges and not about open data, which this section is about.

 Page 12, there is a number “about 9,00,000 ASHAs…” and it is not clear which number this is.

 The authors should state if artificial intelligence was used for any purpose during either the analytical work or writing of the manuscript.

 The manuscript is important locally to India and surrounding countries, due to the publications that were analyzed, but problems and challenges that the authors mention can be applied to many other countries, especially low and middle income countries.

Are the rationale for, and objectives of, the Systematic Review clearly stated?

Yes

Is the statistical analysis and its interpretation appropriate?

Yes

Are sufficient details of the methods and analysis provided to allow replication by others?

Yes

Are the conclusions drawn adequately supported by the results presented in the review?

Yes

Reviewer Expertise:

veterinary science, One Health.

I confirm that I have read this submission and believe that I have an appropriate level of expertise to confirm that it is of an acceptable scientific standard, however I have significant reservations, as outlined above.

# Response

**Published:** 2026-01-19

**Authors:** NAHEEDA HAMZA

**Affiliations:** Centre for Medical Ethics, University of Oslo, Oslo, Norway

Dear Sara Savic, Thank you very much for your valuable time and patience in reviewing our manuscript. We have now revised the manuscript by incorporating all your comments. With regard to your comment on page 10, although the challenges described by Dahal et al are framed as One Health challenges, they are also highly relevant to data sharing. For this reason, we have included them under the section on challenges in data sharing. Regarding your comment on page 12, we intended to indicate that there are approximately 900,000 (nine hundred thousand) Accredited Social Health Activists in India. This has now been clarified in the manuscript. We have also provided a summary of the revisions made in the latest version. Please let us know if you have any further comments or suggestions. Thank you once again for your constructive feedback.

# Reviewer report: Reviewer response for version 1

**DOI:** 10.21956/openreseurope.22807.r65782

**Published:** 2026-01-02

**Authors:** Donal O` Mathuna

**Affiliations:** 1 The Ohio State University, Ohio, USA

This is an excellent article. The methods were carried out appropriately. The thematic analysis was very insightful. The findings are reported clearly and written up very well.

 Under eligibility criteria (p. 4), it would be good to provide a justification for limiting the search to 10 years. The Manhattan Principles for OH were published in 2004 and Open Science is similarly more than 10 years in existence.

 The inclusion criterion for published “in India” (p. 4) has the potential to exclude articles by Indian authors or about India which were published somewhere other than in India. However, the search strategies suggest that articles referring to India, regardless of where published, would have been identified. Some clarification of how the “in India” criterion was implemented should be added and also discussion about the impact of this criterion and its potential implications should be added.

 On p. 10, the authors say that open sharing of data saves time and money “by preventing researchers” from duplicating studies. Prevention seems too strong a term as research often is replicated in spite of OS. This approach may encourage unneeded replication but hardly prevents it.

 A few typos should be corrected.

 On page 9, “the lack of skilled personals” should be “the lack of skilled personnel”.

 On p. 10, the space after humans in “humans , animals” should be removed.

 On p. 14, and is missed after low- in “Moreover, in a low- middle-income country”.

Are the rationale for, and objectives of, the Systematic Review clearly stated?

Yes

Is the statistical analysis and its interpretation appropriate?

Not applicable

Are sufficient details of the methods and analysis provided to allow replication by others?

Yes

Are the conclusions drawn adequately supported by the results presented in the review?

Yes

Reviewer Expertise:

One Health research ethics; systematic reviewing

I confirm that I have read this submission and believe that I have an appropriate level of expertise to confirm that it is of an acceptable scientific standard.

# Response

**Published:** 2026-01-19

**Authors:** NAHEEDA HAMZA

**Affiliations:** Centre for Medical Ethics, University of Oslo, Oslo, Norway

Dear Prof. Donal, Thank you very much for your time and patience in reviewing our manuscript. We have incorporated all your suggestions in the latest version. We sincerely thank you for your valuable input, which has improved the clarity of our work.

# Reviewer report: Reviewer response for version 1

**DOI:** 10.21956/openreseurope.22807.r61811

**Published:** 2025-10-25

**Authors:** Joseph Mwanzia Nguta

**Affiliations:** 1 University of Nairobi, Nairobi, Nairobi, Kenya

The article explores challenges and opportunities in integration of Open science into One Health research. Findings will guide policy and practice towards ethical conduct of research at the human, animal, environmental interface. The introduction is well written. Methods used to respond to the study questions are well articulated to guide reproducibility of the scoping review. I recommend indexing of the article.

Are the rationale for, and objectives of, the Systematic Review clearly stated?

Yes

Is the statistical analysis and its interpretation appropriate?

Yes

Are sufficient details of the methods and analysis provided to allow replication by others?

Yes

Are the conclusions drawn adequately supported by the results presented in the review?

Yes

Reviewer Expertise:

One Health research ethics; One Health research; Ethics of One Health research

I confirm that I have read this submission and believe that I have an appropriate level of expertise to confirm that it is of an acceptable scientific standard.

# Response

**Published:** 2025-10-28

**Authors:** NAHEEDA HAMZA

**Affiliations:** Centre for Medical Ethics, University of Oslo, Oslo, Norway

Dear Dr. Joseph Mwanzia Nguta,

 Many thanks for your valuable time and patience in reviewing our manuscript. We also sincerely appreciate your kind recommendation.

 Kind regards,

 Naheeda Hamza
